# Atlanta and the Yellow Jack

**Published:** 1897-10

**Authors:** 


### Editorial.

### ATLANTA AND THE YELLOW JACK.

The attitude of Atlanta' as evidenced in the invitation of welcome extended to refugees from all the yellow fever infected districts, is one of serene complaisance and security. There are already about two thousand citizens of New Orleans, Mobile and the smaller towns where the disease has appeared, who have fled from their homes and have found an asylum here. This number is being daily increased as the disease spreads and continues.

Just how far this feeling of security is founded on fact remains to be seen. The fact that yellow fever has never gained a foothold here is not sufficient proof that it never will. It establishes nothing beyond a probability against an epidemic. There is nothing topographically or climatically to substantiate the belief that the disease can not exist here, beyond the fact that Atlanta is inland and has an elevation of 1050 feet. Yellow fever is a disease of the seacoast and infrequently prevails in regions with an attitude of over 1000 feet, but it has been known to flourish in the interior of Venezuela at an elevation of 8000 feet.

The Board of Health has very properly officially protested against the current opinion that this city is immune, and has pointed out the danger to life, and business interests incurred by the emptying of the infected region upon Atlanta.

It is more than probable that a few weeks more will see the last of the fever and the return of the refugees and the subsidence of fear, so that in the present instance it is no great matter for us to be especially agitated over the situation. But in the future it would be the part of wisdom to close our gates against the infection, for the risk is too great and the profits, except to hostelries and railroads, too small to warrant such quixotic philanthropy.

---

Dr. C. M. Blackford, Jr., has resigned his instructorship in bacteriology and pathology at the Atlanta Medical College to accept a professorship in the same branches at the Augusta Medical College. The vacancy in the Atlanta college thus occasioned has not yet been filled.

Dr. Blackford has recently committed matrimony. The hearty good wishes of the *Record* follow him.

*The Georgia Journal of Medicine and Surgery* has been launched in Savannah under the editorship of Drs. St. J. B. Graham, W. E. Dudley, and W. E. Fitch. It presents an excellent appearance typographically, and its columns are well filled with original articles, judiciously selected abstracts, and pointed and well-written editorials.

We wish the new journal much success, and believe that it will achieve it, if it adheres to the aims and purposes set forth in its first number.

---

Students of medicine who are planning to take up study in one of the colleges in Louisville, Ky., during the coming year will be glad to learn that the Young Men's Christian Association, through their college department, are planning for special features that will be of interest to college men. The college department will be able to help students find good places to board and room, and, besides the usual features in the way of receptions and entertainments, the privileges in the central department building, including a new magnificent swimming-pool will be placed at the disposal of students who do not live in Louisville for a nominal fee, which can be paid after examination of the privileges mentioned. Particulars as to board, room and other matter can be secured by address- the college secretary, Mr. E. A. Forbes, 318 W. Broadway, Louisville, Ky.

---

### AMERICAN PEDIATRIC SOCIETY

Is making a collective investigation of infantile scurvy as occurring in North America, and earnestly requests the co-operation of physicians, through their sending of reports of cases, whether these have already been published or not. No case will be used in such a way as to interfere with its subsequent publication by the observer. Blanks containing questions to be filled out will be furnished on application to any one of the committee. A final printed report of the investigation will be sent to those furnishing cases.

[Signed] J. P. CROZER GRIFFITH, M. D.,
*Chairman*, 123 S. 18th St., Phila.
WILLIAM D. BOOKER, M. D.,
853 Park Ave., Baltimore.
CHARLES G. JENNINGS, M. D.,
457 Jefferson Ave., Detroit.
AUGUSTUS CAILLE, M. D.,
753 Madison Ave., New York City.
J. LOVETT MONSE, M. D.,
317 Marlboro St., Boston.
*Committee.*

